# Chemical composition of indoor and outdoor PM_2.5_ in the eastern Arabian Peninsula

**DOI:** 10.1007/s11356-024-34482-5

**Published:** 2024-07-30

**Authors:** Ersin Tutsak, Balint Alfoldy, Mohamed M. Mahfouz, Jassem A. Al-Thani, Oguz Yigiterhan, Imran Shahid, Rima J. Isaifan, Mustafa Koçak

**Affiliations:** 1 Environmental Science Center, Qatar University, Doha, Qatar; 2 Aerosol d.o.o., 1000 Ljubljana, Slovenia; 3 College of Science and Engineering, Hamad Bin Khalifa University, Doha, Qatar; 4 Nicosia, Cyprus

**Keywords:** PM_2.5_, Indoor/outdoor, Chemical composition, Arabian Peninsula

## Abstract

Water-soluble and trace metal species in fine particulate matter (PM_2.5_) were determined for indoor and outdoor environments in Doha, Qatar. During the study period, PM_2.5_ concentrations showed significant variability across several indoor locations ranging from 7.1 to 75.8 μg m^−3^, while the outdoor mass concentration range was 34.7–154.4 µg m^−3^. The indoor and outdoor PM_2.5_ levels did not exhibit statistically significant correlation, suggesting efficient building envelope protection against outdoor PM_2.5_ pollution. Rather than outdoor sources, human activities such as cooking, cleaning, and smoking were the most significant influence on chemical composition of indoor PM_2.5_. NH_4_^+^ concentration was insufficient to neutralize SO_4_^2−^ indoors and outdoors, indicating the predominant presence of NH_4_HSO_4_. The enrichment factors indicated that outdoor Fe, Mn, Co, Cr, and Ni in PM_2.5_ mostly originated from crustal sources. In contrast, the remaining outdoor trace metals (Cu, Zn, As, Cd, Pb, and V) were mainly derived from anthropogenic sources. The indoor/outdoor concentration ratios revealed significant indoor sources for NH_4_^+^ and Cu. The crustal matter, water-soluble ions, and sea salt explained 42%, 21%, and 1% of the indoor PM_2.5_ mass, respectively. The same groups sequentially constituted 41%, 16%, and 1% of the outdoor PM_2.5_ mass.

**Supplementary Information:**

The online version contains supplementary material available at 10.1007/s11356-024-34482-5.

## Introduction

Particulate matter, particularly with a diameter less than 2.5 µm (PM_2.5_), has adverse health effects, which can harm human cardiovascular and respiratory systems as well as the immune function (Lippmann 2014; Glencross et al. 2020; Yin et al. 2020). According to the World Health Organization (WHO), ambient air pollution was responsible for an estimated 4.2 million premature deaths worldwide in 2019. Therefore, accurately assessing air pollution levels is crucial to evaluating health effects and associated costs. The impact of particulate matter on public health depends on its concentrations, chemical composition, particle size distribution, and superficial area (Valavanidis et al. 2008; Okuda 2013). Nonetheless, the chemical composition mainly determines the toxicity level of particulate matter (Park et al. 2018). Therefore, particulate matter mass concentrations and chemical composition should be evaluated together to explain their impacts on human health.

The Arabian Peninsula’s air quality status is affected by various natural and anthropogenic emissions and meteorological conditions that generate complex temporal and spatial gradients. In addition to local pollution, the region is also affected by pollution transported from distant sources. Lelieveld et al. (2009) demonstrated the influence of long-range transport of air pollution from Europe and the Middle East and natural emissions in the region. Desert dust is the primary natural source of particulate matter over the Arabian Peninsula. However, Osipov et al. (2022) carried out ship-borne measurements in the Arabian Peninsula. Their modeling results exhibited that hazardous fine particulate matter is mainly of anthropogenic origin (> 90%) and is distinctly different from that of less hazardous coarse desert dust particles. They showed that desert dust dominates both the fine and coarse aerosol size fractions, masking the human signal since most (53%) of the aerosol optical depth was caused by human-made particles. Accordingly, anthropogenic air pollution is a primary health concern in the Arabian Peninsula (Isaifan 2023). On the other hand, indoor particulate matter levels and their chemical composition are essential knowledge in regions such as Qatar, where people spend most of their time indoors, especially during hot days. People’s welfare is tied to the quality of their living conditions, and as living standards rise, so do people’s expectations for a healthy living environment. Considering these facts, this study aims to explore the chemical compositions of PM_2.5_ indoor and outdoor mass concertation and their correlation in order to assist environmental regulators and policymakers in formulating recommendations and guidelines to safeguard the population from the adverse health effects of air pollution in Qatar.

## Materials and methods

### Sampling site and aerosol sampling

Qatar is a peninsula situated on the northeast coast of the Arabian Peninsula, bordered by Saudi Arabia from the south and surrounded by the Arabian Gulf on the east, north, and west. The country’s surface area is 11,586 km^2^ and its population stands at ~ 3 million as reported in 2023. The population has increased 1.5 times over the past 10 years. Doha, the capital city of Qatar, is bordered by industrialized areas 30 km to the north (Mesaieed) and 60 km to the south (Ras Laffan), where most of Qatar’s oil and gas industry is situated. Furthermore, 12 km to the southwest, an industrial area with big smelting workshops is located. Apart from local emission sources, the city of Doha is also affected by air masses reaching from the Arabian Peninsula and the Arabian Gulf. These air masses may carry various natural and anthropogenic aerosols associated with mineral dust intrusions, petroleum industry, shipping activities, and sea spray (Javed et al. 2019; Javed and Guo 2020).

**Fig. 1:**
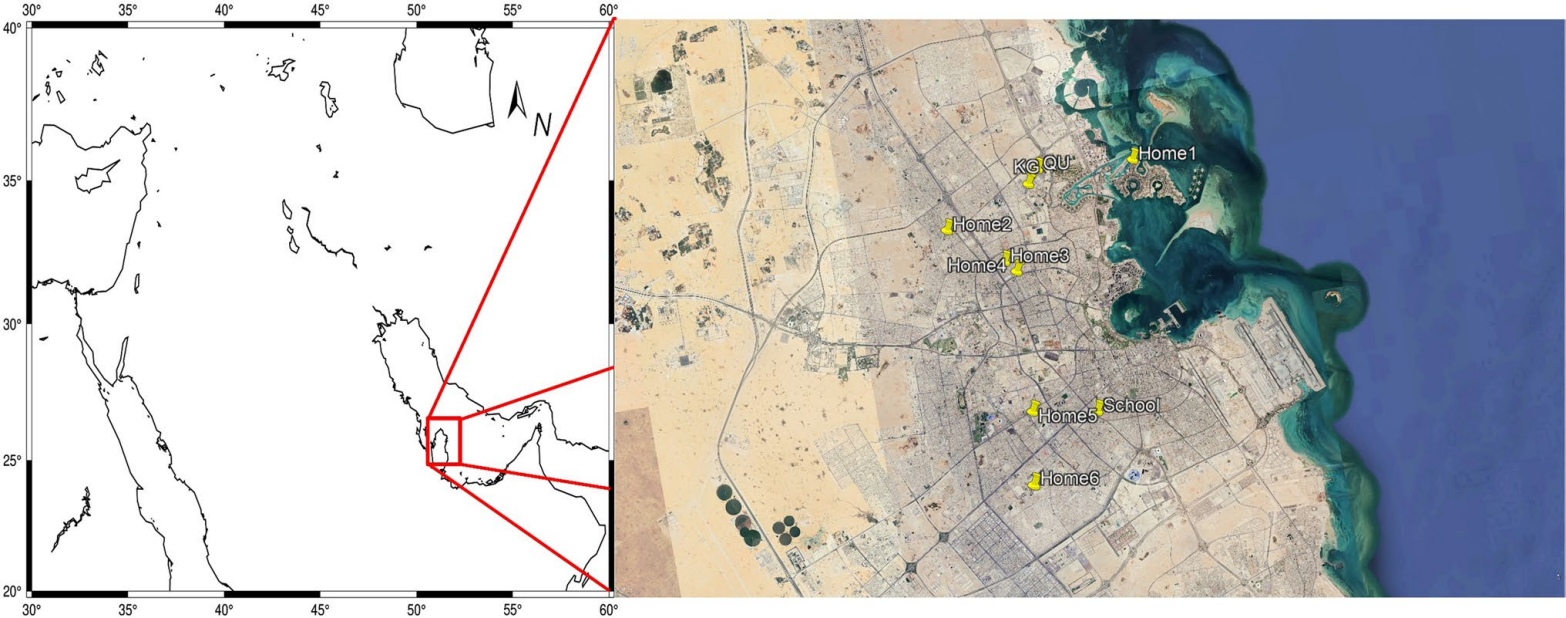
Location of sites where PM_2.5_ samples were collected

Indoor and outdoor PM_2.5_ samples were simultaneously obtained from urban/arid Doha (Qatar) during two sampling campaigns that cover winter (from December to March) and transition (October–November and April–May) seasons. The first campaign commenced on 12 February and finished on 27 May 2018, while the second was carried out from 27 September to 3 November 2018. Correspondingly, a total of 36 and 18 PM_2.5_ samples were collected for winter and transition periods. The sampling period for each sample varied from 2 to 7 days. From February 12 to 14 and October 14 to 18, no outdoor samples were collected due to a mechanical failure of the instrument. Except for Friday and Saturday, residential indoor and outdoor aerosol samples were continuously obtained. In contrast, indoor samplers from classes were exclusively operated for about 8 h a day during the classes throughout the five working days of the week. PM_2.5_ sampling strategy designed (see Table 1) to reflect indoor air quality, including five homes (one of them being a smoker’s home), four classrooms, a kindergarten reception area, laboratory, Qanat Quartier (seaside), and their corresponding outdoor samples (Fig. 1). Throughout the study period, indoor environment windows were kept closed.

**Table 1:** Categorization of indoor and outdoor samples obtained from urban/arid Doha during 12 February–27 May 2018 and 27 September–3 November 2018

Site	Indoor	Indoor	Outdoor
Kindergarten	Class 1	Reception	Garden
School	Class 2	Class 3	Garden
Qanat Quartier seaside	Home 1	-	Balcony
Home-smoker	Home 2	-	Garden
Home-nonsmoker	Home 3	-	Garden
Home-nonsmoker	Home 4	-	Garden
Home-nonsmoker	Home 5	-	Garden
Home-nonsmoker	Home 6	-	Garden
Home-nonsmoker	Home 7	-	Garden
Qatar University	Class 4	Laboratory	Garden

Indoor and outdoor PM_2.5_ aerosol sampling was simultaneously carried out by a volume Harvard impactor with a size-selective inlet. The instrument was operated at 10 L per minute in order to ensure a cutoff diameter of 2.5 µm with an efficiency of 50%. The atmospheric particles were collected on Teflon filters (Pall, Teflon Membrane Disc Filters, 37 mm diameter and 2-µm pore size). The filters were conditioned in desiccator chambers under controlled temperature and relative humidity (*T* = 20 °C and RH = 40%) before and after the sampling. The mass of the filters was determined gravimetrically by a Mettler Toledo microbalance with a 0.1 µg readability. Before weighing, the static electrical charge of filters was neutralized by applying a Milty Zerostat 3 antistatic gun. The weight difference between the loaded and empty filters (net mass) was divided by the sampled air volume to calculate PM_2.5_ masses for indoor and outdoor samples.

### Analysis of elements and water-soluble ions

Half of the filters were extracted using 5 mL of HNO_3_ (65% nitric acid) and 2 mL of HF (40% hydrofluoric acid). A total digestion procedure followed by inductively coupled plasma mass spectrometry (ICP-MS) was performed to measure elemental concentrations of Cr, Mn, Fe, Co, Ni, Cu, Zn, Al, V, As, Cd, and Pb in the aerosol samples, following the method described in detail by Yilmaz (2016). The detection limits (LOD), calculated as three times the standard deviation (3*σ*) of blanks, were 0.01 ng/m^3^ for Cd, V, As, and Co; 0.2 ng/m^3^ for Pb, Ni, Cu, Mn, and Cr; 10 ng/m^3^ for Zn and Fe; and 10 ng/m^3^ for Al. Analytical recoveries were better than 85% for Fe, Co, Ni, Cu, Cr, and As and higher than 90% for Al, Mn, Zn, Cd, Pb, and V. Blank contributions were found to be less than 8%.

In order to measure water-soluble ions (Cl^−^, NO_3_^−^, SO_4_^2−^, Na^+^, NH_4_^+^, K^+^, Mg^2+^, and Ca^2+^), half of the filters were transferred into 20 mL vials, and 10 mL ultrapure Milli Q water (18.2 Ωm) was added in a laminar flow hood to avoid possible contamination. Samples were extracted for 60 min using a mechanic shaker, and after removing filters, 100 µL of chloroform was added to each bottle to prevent any biological activity. Before analysis, aliquots of samples were filtered by a nylon membrane syringe filter (0.45 µm; 25 mm diameter; VWR International). Water-soluble ions in PM_2.5_ samples were analyzed by ion chromatography instrument (ICS-5000). Water-soluble cations (Na^+^, NH_4_^+^, K^+^, Mg^2+^, and Ca^2+^) were determined by using CS12A separation column, 20 mM MSA eluent, DRSC600 suppressor, and conductivity detector. Meanwhile, water-soluble anions (Cl^−^, NO_3_^−^, SO_4_^2−^) were measured by using an AS-11 separation column, 12 mM NaOH eluent, DRSA600 suppressor, and conductivity detector as per the method reported in (Bardouki et al. 2003; Nehir and Koçak 2018). The detection limits for water-soluble ions were better than 1 ppb, and the blank contributions for all water-soluble ions were found to be less than 9%. Concentrations were corrected accordingly. The non-sea-salt fraction of water-soluble ions was determined using the following equation: nss-Ion = (Ion)_aerosol_ − (Ion/Na^+^) _seawater_ × (Na^+^) _aerosol_. In this study, mass ratios of 0.119, 0.037, and 0.251 were used for (Mg^2+^/Na^+^)_seawater_, (K^+^/ Na^+^)_seawater_, and (SO_4_^2−^/ Na^+^)_seawater_, respectively (Millero 2006).

### Calculation of the enrichment factor for trace elements

The enrichment factor (EF) was employed for distinguishing between natural and anthropogenic sources of trace metals. The EF is typically defined by the following equation:

$$\text{EF}={\left(\left[E\right]/\left[R\right]\right)}_{\text{sample}}/{\left(\left[E\right]/\left[R\right]\right)}_{\text{crust}}$$

where *E* represents the element of interest and *R* is the reference element for crustal material. ([*E*]/[*R*])_sample_ is the concentration ratio of *E* to *R* in the aerosol sample, and ([*E*]/[*R*])_crust_ is the average concentration ratio of *E* to *R* in the Earth’s crust. In this study, Al was chosen as the reference element. The crustal ratios of the elements were obtained from Rudnick and Gao (2014). A trace metal in particulate matter is usually considered to have a significant crustal source if its crustal enrichment factor (EF) value is less than 10; on the other hand, a substantial portion of an element is thought to have a non-crustal source if its EF value is greater than 10 (Chester et al. 1999).

### Statistical analysis

Data were statistically analyzed using SPSS 28 software, which included performing Pearson correlation analysis and principal component analysis with varimax rotation.

## Results and discussions

### *PM*_*2.5*_* mass*

**Fig. 2:**
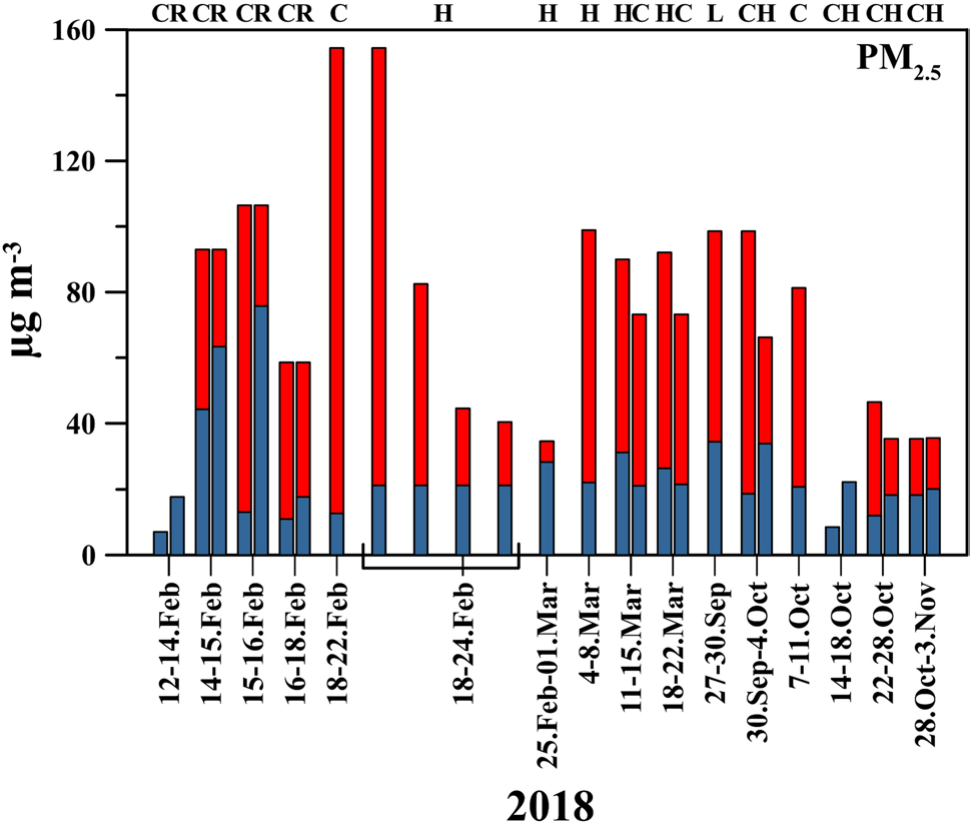
Temporal variability of indoor (blue bar) and outdoor (red bar) PM_2.5_. Explanation of abbreviation: Class (C), Home (H), Lab (L), and Reception (R)

Table 2 shows the arithmetic mean concentrations of PM_2.5_ along with the standard deviation for indoor and outdoor environments. Throughout the sampling campaign, indoor mass concentrations varied between 7.1 and 75.8 µg m^−3^ (Fig. 2) with a mean value of 23.9 ± 16.3 µg m^−3^. Indoor PM_2.5_ concentrations were found to be decreasing in the order Reception > Home > Classroom; 41.1 ± 33.5 µg m^−3^, 25.1 ± 5.8 µg m^−3^, and 14.4 ± 5.9 µg m^−3^, respectively, as can be shown in Table 2. In comparison, the outdoor PM_2.5_ concentrations exhibited large variability ranging from 34.7 to 154.4 µg m^−3^ (Fig. 2) with an arithmetic mean of 76.9 ± 29.0 µg m^−3^. The degree of relationship between indoor and outdoor pollutant levels has been attributed to infiltration rates, decay rates, and emissions from indoor sources (Meier et al. 2015). Except for the Reception site, the indoor and outdoor PM_2.5_ levels did not show statistically significant correlation coefficients, suggesting efficient building envelope protection against outdoor PM_2.5_ pollution. The lowest I/O (0.08) ratio was observed during a dust event, and the highest ratio was monitored for smoker inhabitants with a value of 0.82, denoting the influence of smoking on indoor air quality. Correspondingly, the highest and lowest I/O were observed for Reception (0.53) and Classrooms (0.18) sites, followed by the Home (0.32) sites. Frequent door openings caused more exchange between the Reception site and the outdoor environment, resulting in higher PM_2.5_ levels in the Reception site.

**Table 2:** Statistical summary of indoor and outdoor PM_2.5_ (μg m^−3^)

PM_2.5_	Indoor	Outdoor	I/O
All	23.9 ± 16.3	76.9 ± 29.0	0.31
Classroom	14.4 ± 5.9		0.18
Reception	41.1 ± 33.5		0.53
Home	25.1 ± 5.8		0.32

On all sampling days, the outdoor concentrations of PM_2.5_ exceeded the WHO recommended limit for PM_2.5_ (15 μg m^−3^ for 24-h mean as per the 2021 standards). Saraga et al. 2017 reported the overall mean concentrations of 15.5 μg/m^3^ and 172 μg/m^3^ for indoor and outdoor PM_2.5_, respectively, for the study period from April to June 2015 in Qatar. The present outdoor PM_2.5_ average mean concentration was 2.2 times less than Saraga et al. (2017) reported. This discrepancy might be attributed to the dust events frequently occurring over the region during the spring and summer seasons (Farahat 2016). If the Reception site is not considered, this reported average indoor PM_2.5_ in this study would generally agree with the indoor values reported in Saraga et al. (2017).

### Water-soluble ions

Table 3 shows a statistical summary of the water-soluble ions attained from this study, including arithmetic mean concentrations and their standard deviations and minimum and maximum values. The indoor water-soluble ions were in the order SO_4_^2−^ > NH_4_^+^  > Ca^2+^  > NO_3_^−^ > Cl^−^ > Na^+^  > Mg^2+^  > K^+^, while the outdoor ions followed the decreasing order SO_4_^2−^ > Ca^2+^  > NH_4_^+^  > NO_3_^−^ > Cl^−^ > Na^+^  > Mg^2+^  > K^+^. The highest average indoor and outdoor concentrations were found for SO_4_^2−^, with values of 2.92 μg m^−3^ and 7.87 μg m^−3^, respectively.

**Table 3:** Statistical summary for the water-soluble ions in PM_2.5_ (μg m^−3^)

	Indoor			Outdoor		
Variable	Mean ± std	Minimum	Maximum	Mean ± std	Minimum	Maximum
Na^+^	0.05 ± 0.04	0.01	0.16	0.18 ± 0.10	0.06	0.40
NH_4_^+^	0.72 ± 0.47	0.20	2.07	1.17 ± 0.88	0.25	2.80
K^+^	0.02 ± 0.01	0.00	0.04	0.06 ± 0.03	0.02	0.15
Mg^2+^	0.03 ± 0.02	0.00	0.12	0.11 ± 0.05	0.05	0.23
Ca^2+^	0.69 ± 0.95	0.08	4.40	2.23 ± 1.06	0.64	4.38
Cl^−^	0.12 ± 0.10	0.03	0.39	0.24 ± 0.22	0.04	0.73
NO_3_^−^	0.18 ± 0.12	0.04	0.51	0.69 ± 0.55	0.08	1.85
SO_4_^2−^	2.92 ± 1.95	0.51	7.86	7.87 ± 4.82	2.55	19.67

It was followed by NH_4_^+^ indoors with a mean value of 0.72 ± 0.47 μg m^−3^, while Ca^2+^ was found to be the second highest outdoors with a mean of 2.23 ± 1.06 μg m^−3^. The most significant ions were SO_4_^2−^ (62% indoor; 63% outdoor), NH_4_^+^ (15% indoor; 10% outdoor), Ca^2+^ (14% indoor; 17% outdoor), and NO_3_^−^ (4% indoor; 5% outdoor) contributing 95% to the total ion mass. The contributions of Cl^−^, Na^+^, Mg^2+^, and K^+^ to indoor and outdoor ion mass were small of only 2%, 1%, 1%, and 1%, respectively. The water-soluble ion concentrations in our PM_2.5_ samples were lower than those previously reported in Qatar (Saraga et al. 2017; Javed and Guo 2020). For instance, the values of 4.82 μg m^−3^ and 15.24 μg m^−3^ were reported by Saraga et al. (2017) for indoor and outdoor SO_4_^2−^, respectively. This difference may be attributed to the length of the sampling campaigns.

**Fig. 3:**
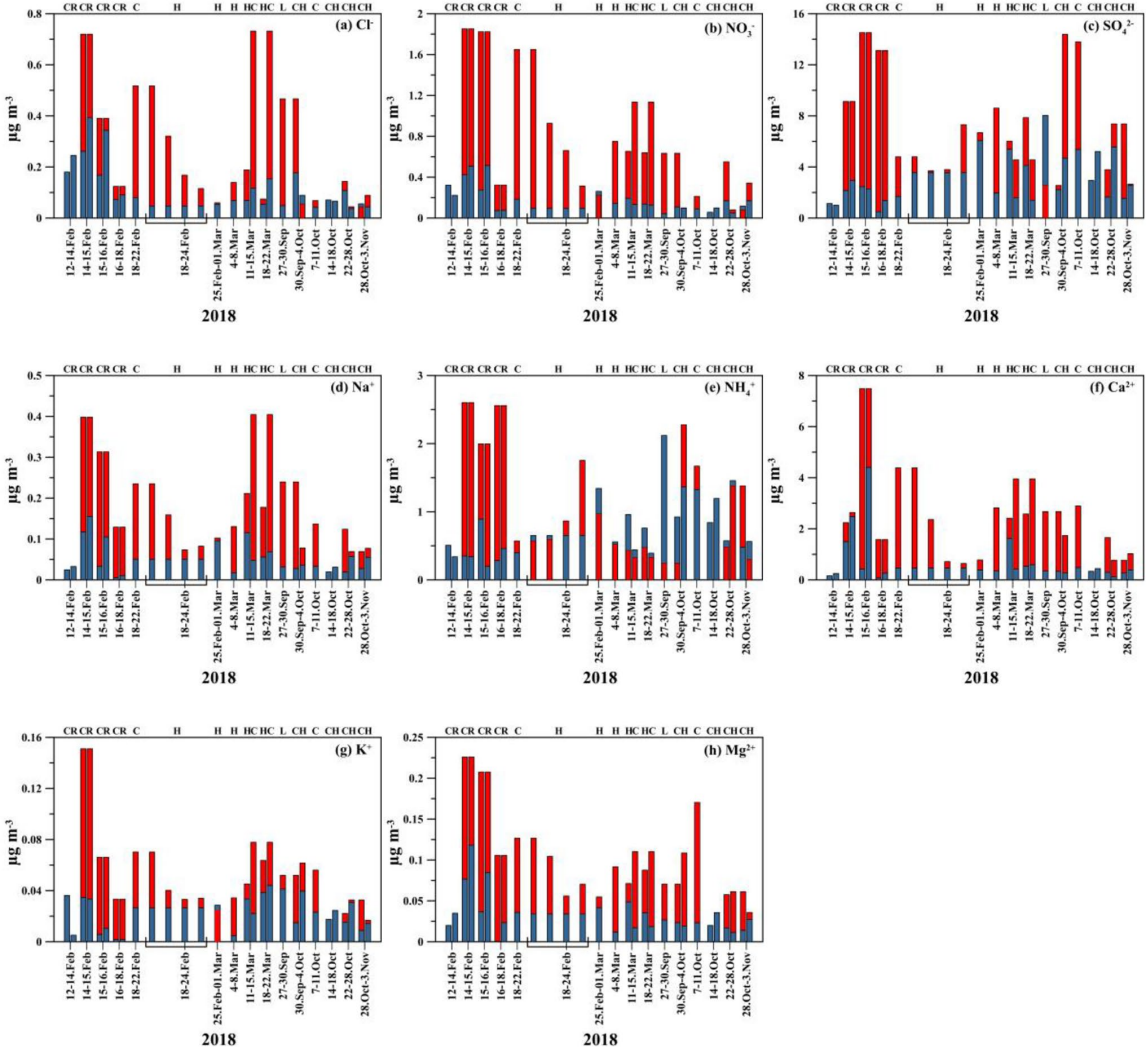
Temporal variability of indoor (blue bar) and outdoor (red bar): **a** Cl^−^, **b** NO_3_^−^, **c** SO_4_^2−^, **d** Na^+^, **e** NH_4_^+^, **f** Ca^2+^, **g** K^+^, and **h** Mg.^2+^ in PM_2.5_. Explanation of abbreviation: Class (C), Home (H), Lab (L), and Reception (R)

**Fig. 4:**
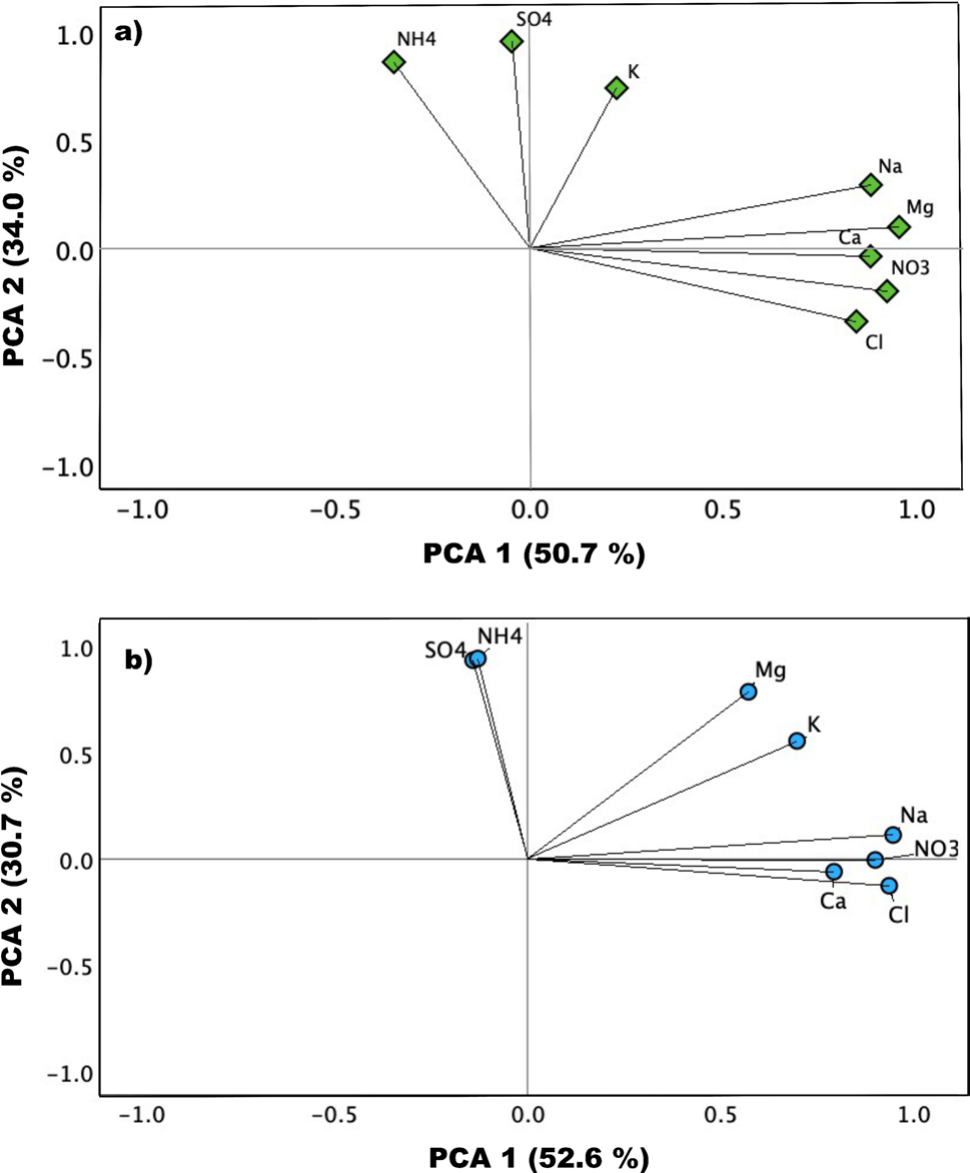
Principal component analysis (PCA) biplots for **a** indoor and **b** outdoor ions in PM_2.5_, based on the first two principal components, PCA1 and PCA2

The temporal variations of indoor and outdoor water-soluble ion concentrations throughout the sampling periods are depicted in Fig. 3. The results revealed significant fluctuations in the daily concentrations of water-soluble ions. This type of variability suggested the influence of various sources of the observed levels. Table 1S presents the indoor and outdoor correlations between water-soluble ions. As anticipated, sea salt species (Na^+^, Cl^−^ and Mg^2+^) showed high correlation in both indoor and outdoor, indicating they were mainly originated from sea salt. NH_4_^+^ was strongly correlated with SO_4_^2−^ (*r* = 0.91 for indoor, *r* = 0.83 for outdoor), demonstrating the formation of ammonium sulfate. Nevertheless, the analyzed regression revealed that NH_4_^+^ is insufficient to neutralize SO_4_^2−^. This might be related to the main presence of NH_4_HSO_4_ instead of (NH_4_)_2_SO_4_ (Koçak et al. 2004). The correlation between Ca^2+^ and NO_3_^−^ was significant indoors and outdoors, indicative of the existence of Ca(NO_3_)_2_. Figure 4a, b shows the biplot of PCA for indoor and outdoor trace metals. PCA results showed that the first two components accounted for about 83.3% (indoor) and 84.7% (outdoor) of the total variance. PC1 and PC2 explained 52.6% (indoor) and 30.7% (indoor) and 50.7% (outdoor) and 34.0% (outdoor) of the total variance, respectively. Specifically, Cl^−^, NO_3_^−^, Na^+^, Mg^2+^, and Ca^2+^ played significant roles in PC1, with loading coefficients greater than 0.65. The effects of SO_4_^2−^ and NH_4_^+^ were significant in PC2, with loading coefficients exceeding 0.90. The main sources of the ions in PC1 for both indoor and outdoor were natural sources (crustal and sea salt), whereas those in PC2 were related to gas to particle secondary aerosols sources.

### Trace metal species

**Fig. 5:**
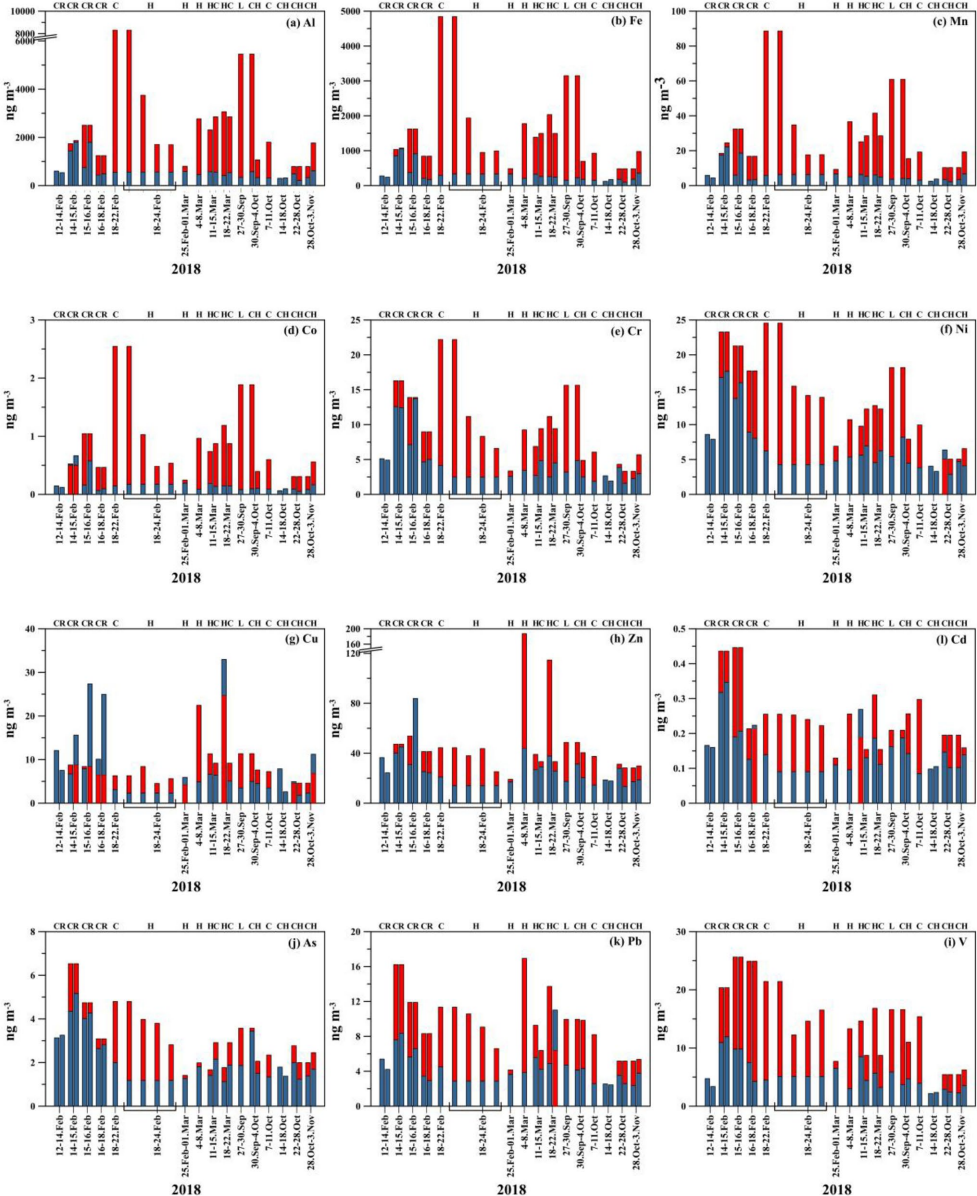
Temporal variability of indoor (blue bar) and outdoor (red bar) trace metals **a** Al, **b** Fe, **c** Mn, **d** Co, **e** Cr, **f** Ni, **g** Cu, **h** Zn, **i** Cd, **j** As, **k** Pb, and **l** V in PM_2.5_. Explanation of abbreviation: Class (C), Home (H), Lab (L), and Reception (R)

Figure 5 shows the indoor and outdoor temporal fluctuations in the concentrations of metals in PM_2.5_ throughout the study period. Table 4 presents a static summary of trace metal species. The dominant trace metals in PM_2.5_ were Al (612 ± 416 ng m^−3^), Fe (314 ± 243 ng m^−3^), and Zn (27.8 ± 13.4 ng m^−3^) indoors, whereas Al (2696 ± 1907 ng m^−3^), Fe (1545 ± 1064 ng m^−3^), and Zn (49.5 ± 37.9 ng m^−3^) were predominant in outdoors. It is apparent from the results that crustal metal species, namely, Al and Fe, exhibited higher concentrations, comprising 93% and 97% of the measured indoor and outdoor trace metal masses, respectively. In the indoor environment, the trace metals exhibited the following descending order: Al > Fe > Zn > Cu > Ni > Mn > V > Cr > Pb > As > Co > Cd. Correspondingly, the outdoor trace metals followed the decreasing order of Al > Fe > Zn > Mn > V > Ni > Cr > Pb > Cu > As > Co > Cd. The levels of outdoor trace metal concentrations were consistent with the findings of the previous study in Qatar (Javed and Guo 2020). Furthermore, they reported similar arithmetic mean concentrations for Co, Cd, and Pb. In contrast, the mean concentrations of crustal species such as Al, Fe, and Mn were inconsistent with those observed in our study. This disparity could mainly be attributed to variations in the strength of dust emissions during the respective sampling periods.

**Table 4:** Statistical summary for the metal species in PM_2.5_ (ng m^−3^)

	Indoor			Outdoor		
Variable	Mean ± std	Minimum	Maximum	Mean ± std	Minimum	Maximum
Al	612 ± 416	211	1804	2696 ± 1907	791	8310
Fe	314 ± 243	94	1055	1545 ± 1064	483	4843
Mn	6.3 ± 5.0	2.3	22.2	29.8 ± 19.8	9.3	88.6
Co	0.17 ± 0.16	0.05	0.67	0.86 ± 0.56	0.25	2.55
Cr	4.6 ± 3.3	1.6	13.7	9.5 ± 5.0	3.3	22.2
Ni	7.3 ± 4.2	2.9	17.6	13.6 ± 5.6	5.0	24.5
Cu	8.7 ± 8.0	1.8	33.0	9.0 ± 5.5	4.2	24.7
Zn	27.8 ± 14.6	13.4	83.9	49.5 ± 37.9	19.1	187.2
V	5.3 ± 2.8	2.2	11.9	15.2 ± 5.7	5.4	25.6
As	2.3 ± 1.1	1.1	5.2	3.0 ± 1.4	1.4	6.5
Pb	4.5 ± 2.0	2.4	11.0	9.1 ± 3.7	4.0	16.9
Cd	0.16 ± 0.07	0.16	0.35	0.23 ± 0.09	0.11	0.45

The correlation coefficients for trace metal species are presented in Table S2a and S2b for indoor and outdoor particles, respectively. As expected, there was a strong correlation between the crustal species (Al, Mn, and Fe) outdoors. Additionally, Co and Cr were strongly correlated with crustal species outdoors, while Ni was moderately correlated. This implies that a substantial proportion of the measured outdoor concentrations of these Co, Cr, and Ni elements were associated with the crustal materials. On the other hand, the strength of the correlations between crustal species and all other trace metals, excluding Cu, increased significantly in the indoor environment compared to the outdoor environment. This may be attributed to the resuspension of the particles from the floor surfaces due to active human activities such as walking and vacuum cleaning (Abt et al. 2000; Wang et al. 2021). The lower increment in the indoor correlations of Cu with crustal species may imply a different indoor source for Cu (please see the “Relationship between indoor and outdoor for measured species” section).

**Fig. 6:**
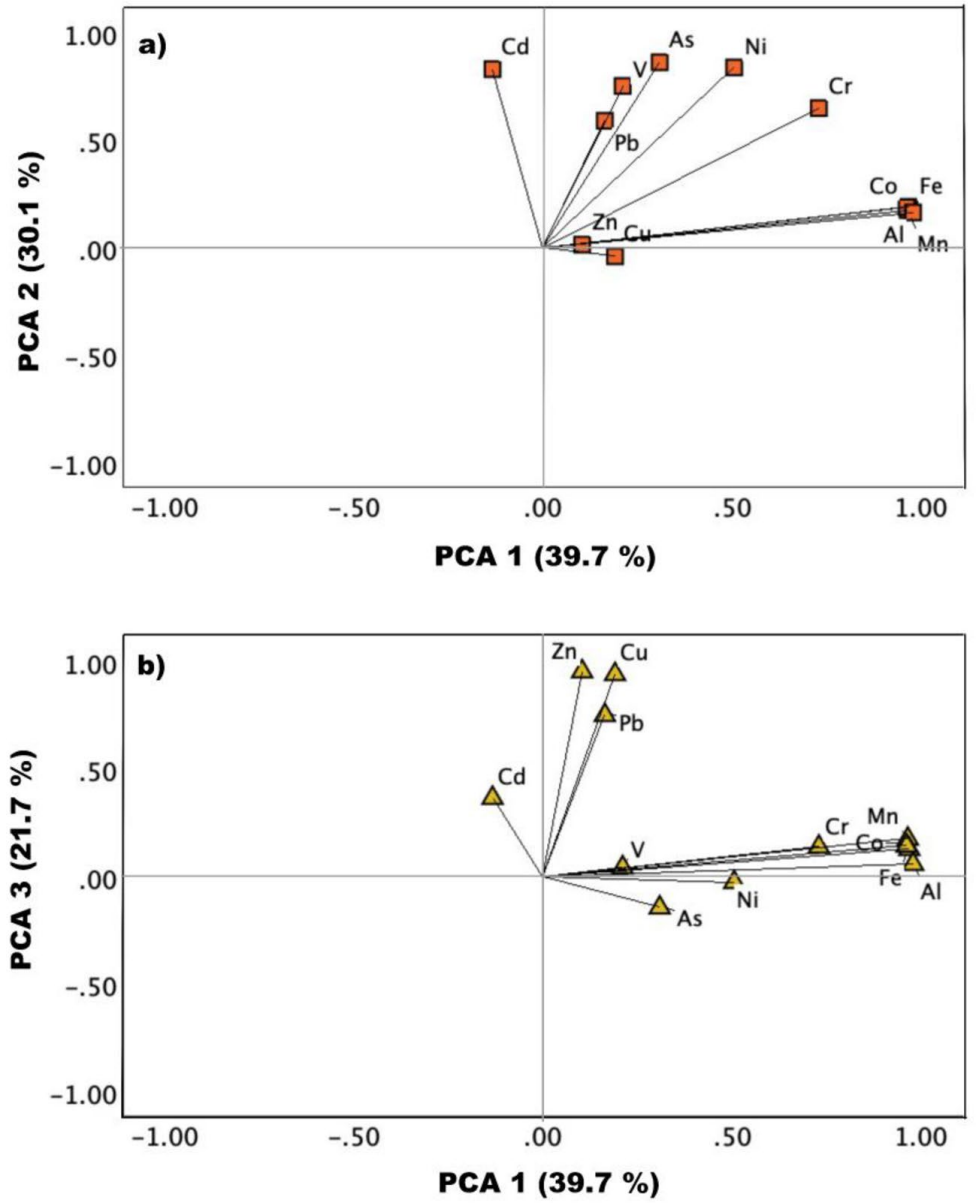
Principal component analysis (PCA) biplots for outdoor metals in PM_2.5_, based on **a** PCA 1 and PCA 2 and **b** PCA 1 and PCA 3

Figure 6a, b shows the biplot of PCA for outdoor trace metals. PCA results indicated that the first three components accounted for approximately 91.5% of the total variance. Specifically, PC1, PC2, and PC3 explained 39.7%, 30.1%, and 21.7% of the total variance, respectively. In PC1, Mn, Fe, Co, Al, and Cr had a significant effect, with loading coefficients greater than 0.73. These elements in aerosols mainly come from crustal sources. PC2 was related to Ni, V, As, and Cd, with loading coefficients above 0.90, representing emissions from heavy oil combustion and industrial activities (Masiol et al. 2017; Squizzato et al. 2017). Ni and V are mainly derived from shipping emissions if the V/Ni ratio in PM_2.5_ is greater than 0.7 (Zhang et al. 2014). The average outdoor V/Ni ratio from our study was 1.16 ± 0.29, indicating a significant contribution from shipping emissions. PC3 was associated with Cu, Zn, and Pb, with loading coefficients above 0.75. Cu, Zn, and Pb are indicators of traffic emissions, including brake, tire, and road wear, exhaust, and road dust (Tan et al. 2014; Crilley et al. 2017).

PCA results for indoor trace metals indicated that only one component could be extracted. Except for Cu, all metal species played a significant role in this component, with loading factors higher than 0.65, explaining 75% of the total variance.

**Fig. 7:**
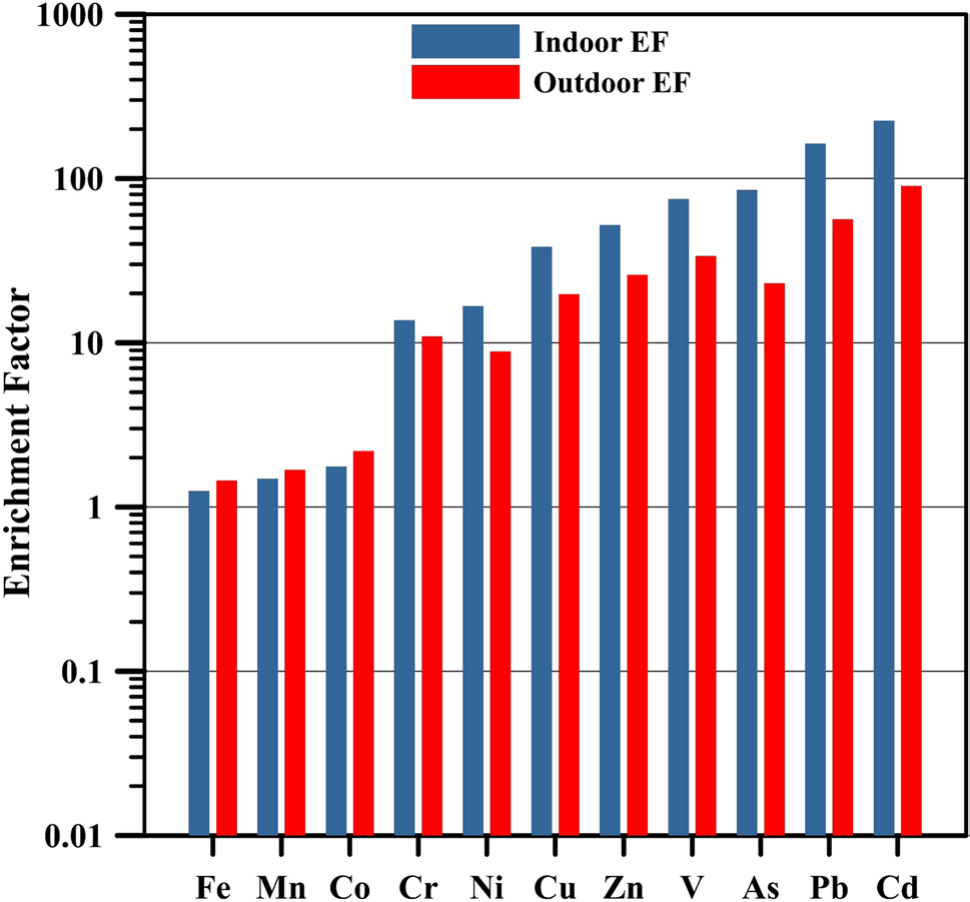
Enrichment factor of trace metal species in indoor and outdoor PM_2.5_ samples

Figure 7 shows EF for indoor and outdoor samples. Outdoor EF values for Fe, Mn, and Co were less than 2, while for Cr and Ni, they were approximately equal to 10, implying that these species found in PM_2.5_ mainly originated from crustal sources. However, the outdoor EF values of 10 for Cr and Ni may also suggest significant enrichments compared to the EF values of Fe, Mn, and Co. Additionally, Fe, Mn, and Co indoor EF values remained under 2. In contrast, indoor EF for Cr and Ni exceeded 10, suggesting that anthropogenic sources contributed noteworthy to indoor Cr and Ni concentrations. For the remaining elements, namely, As, Pb, Cd, V, Zn, and Cu, EF values for indoor and outdoor samples were considerably above 10, indicating significant enrichments. It is worth noting that indoor enrichments for all species except Fe, Mn, and Co were higher than outdoor enrichments. Trace metals such as V, Cr, Mn, Fe, Ni, Cu, Pb, and Zn in particulate matter have the potential to be harmful, according to toxicological research (Rönkkö et al. 2018; Huang et al. 2022). WHO (2000) recommended the values of 5 ng m^−3^ and 150 ng m^−3^ for Cd and Mn, respectively, regardless of where these metals are found in PM_2.5_ or PM_10_ as thresholds to avoid harmful impacts. The results obtained from the present study for Cd and Mn were reasonably lower than the recommended values.

### Relationship between indoor and outdoor for measured species

**Fig. 8:**
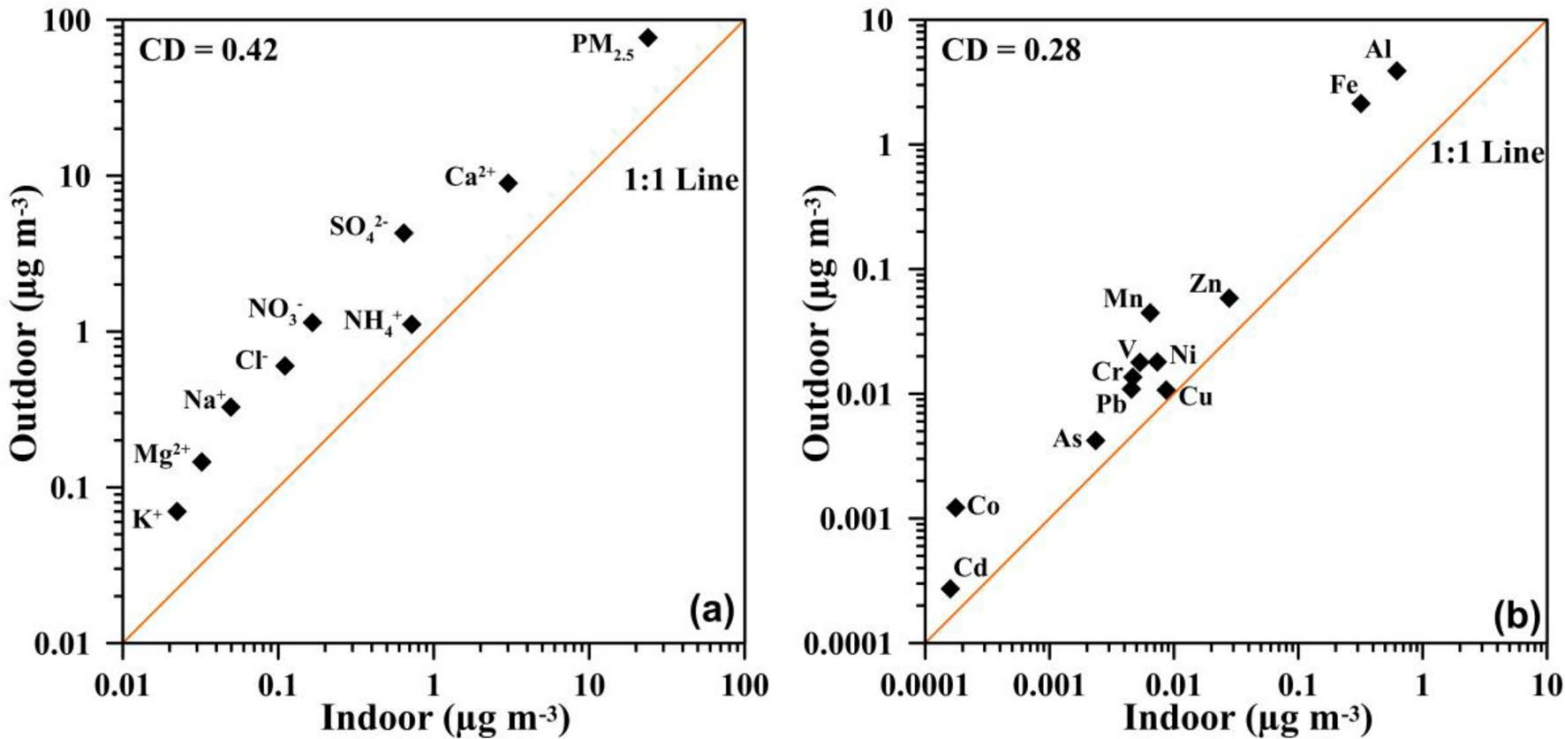
Comparison of average water-soluble ion and elemental metal concentrations between **a** indoor and **b** outdoor environments with the coefficients of divergence

To evaluate spatial variations between indoor and outdoor environments, the coefficients of divergence (CD) for each pair of measured species were computed (Wongphatarakul et al. 1998). When the two sampling environments exhibit similarity, the CD values approach zero. On the other hand, CD values greater than 0.2 indicate spatial heterogeneity (Wilson et al. 2005; Krudysz et al. 2009). Figure 8 shows a lognormal plot of indoor-to-outdoor water-soluble (a) and (b) elemental metal concentrations with the averaged CD values. This study calculated the mean CD values of 0.42 and 0.28 for water-soluble ions and trace metals, respectively. These calculated CD values revealed a significant heterogeneous spatial distribution of water-soluble ions and trace metals between indoors and outdoors. All indoor species concentrations were found to be lower than those in outdoors (Fig. 8). It can be observed that there was a relatively high I/O ratio for NH_4_^+^ and Ca^2+^, indicating the different sources of these two ions (Fig. 8a). The high indoor NH_4_^+^ levels may be related to ammonium salts formed indoors by the reaction between inorganic/organic acids and NH_3_. It was reported that indoor NH_3_ levels are frequently greater than outdoor ones (Ampollini et al. 2019; Li et al. 2020). Indoor ammonia may be emitted from cleaning agents, smoking, building materials, cooking, and the human body through breath, urine, and sweat (Nazaroff and Weschler 2020). For indoor Ca^2+^, sources may be governed by the outdoor resuspension of crustal material from floor surfaces during household activities such as walking and vacuum cleaning or by its enhanced outdoor penetration on high windy days. Trace metals of crustal origin, such as Al, Fe, Mn, and Co, exhibited comparable average I/O ratios, implying common sources (Fig. 8b). In contrast, anthropogenic elements (Cr, As, Pb, Cd, V, Ni, Zn, and Cu) displayed variable I/O values, denoting distinct indoor sources. Notably, Cu demonstrated the highest I/O ratio, suggesting the presence of potential indoor sources. These indoor sources may include emissions generated by indoor electric universal motors found in appliances like vacuum cleaners, food processors, air dryers, and AC units (Szymczak et al. 2007; Hasheminassab et al. 2014; Vicente et al. 2020).

### Mass closure

**Fig. 9:**
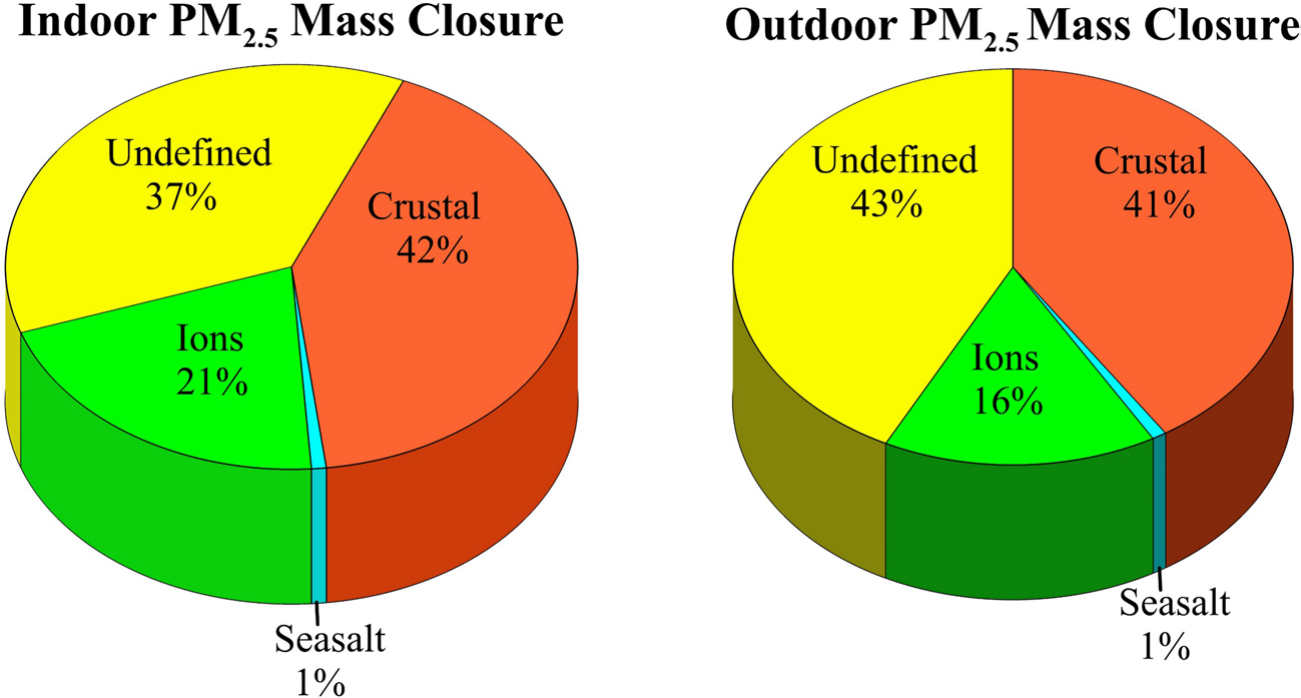
Mass closure analysis for indoor and outdoor PM_2.5_

Chemical mass closure calculations were performed for the indoor and outdoor PM_2.5_. For that purpose, the chemical compositions of indoor and outdoor PM_2.5_ mass were divided into four groups: crustal, sea salt, ions, and an undefined group. The total amount of crustal material was calculated by summing Al_2_O_3_, SiO_2_, CO_3_^2–^, Fe, K, Mg, and Mn (Saraga et al. 2017). Al concentration was multiplied by the factor of 1.89 to obtain Al_2_O_3_, whereas SiO_2_ was found by multiplying derived Al_2_O_3_ by 3. Ca was multiplied by 1.5 to account for CO_3_^2–^. The sea salt contribution was evaluated from Na^+^ concentration, assuming that Na^+^ originates merely from sea salt (Turekian 1976; Koçak et al. 2016). Ionic mass was the sum of nssSO_4_^2−^, NO_3_^−^, NH_4_^+^, nssK^+^, and nssMg^2+^. Figure 9 presents a mass closure analysis for indoor and outdoor PM_2.5_. The figure shows that the crustal material and water-soluble ions were identified as the primary contributors to total particulate mass, with percentages of 42% and 21% indoors and 41% and 16% outdoors, respectively. The ionic mass contribution for indoors increased by 5% compared to the outdoors. The largest ionic contributor to both indoor and outdoor PM_2.5_ mass was SO_4_^2−^ (16% indoor; 13% outdoor), followed by NH_4_^+^ (4% indoor; 2% outdoor). The contribution of sea salt was found to be the smallest, accounting for 1% of the total mass for both indoor and outdoor. The undefined percentage for indoor mass concentration was 37%, while it was 43% for outdoor. These unidentified groups might be mainly attributed to the mass associated with black carbon and organic carbon, which was not measured in this study. The findings of the mass closure results did not align with the previous studies conducted in Qatar by Saraga et al. (2017) and Javed and Guo (2020), who reported 14% and 24% crustal material contributions to outdoor PM_2.5_, respectively. In contrast, a more recent study conducted by Osipov et al. (2022) showed a 52% contribution of mineral dust to PM_2.5_ mass in the region.

## Summary and conclusion

This study revealed indoor and outdoor PM_2.5_ concentrations and its chemical composition in Doha, Qatar, during the winter and transition seasons in 2018. Throughout the study period, there was a notable fluctuation in PM_2.5_ concentrations within indoor locations, ranging from 7.1 to 75.8 μg m^−3^. Outdoor mass concentrations also varied, varying from 34.7 to 154.4 µg m^−3^. Indoor PM_2.5_ levels were mainly governed by indoor sources instead of infiltration from outdoor sources. Concerning improving indoor air quality, air purification and ventilation systems are recommended as possible mitigation strategies. Extended sampling periods are necessary to understand the atmospheric particulate matter environment and climatology in the eastern Arabian Peninsula.

### Supplementary Information

Below is the link to the electronic supplementary material.

Supplementary file1 (DOCX 39 KB)

## Data Availability

The data is available on request from the corresponding author.
