# Supplementary material for: Chemical composition of indoor and outdoor PM2.5 in the eastern Arabian Peninsula
Source: Environ Sci Pollut Res Int. 2024 Jul 30;31(37):49589–600. doi: 10.1007/s11356-024-34482-5 (PMC11324777; doi:10.1007/s11356-024-34482-5)
Supplement: Supplementary file 1 — Supplementary file1 (DOCX 39 KB) [file 11356_2024_34482_MOESM1_ESM.docx]

Supplementary information

**Table S1.** Correlation between water-soluble species in (a) indoor and (b) outdoor aerosol samples.

| **a)** | **Na^+^** | **NH_4_^+^** | **K^+^** | **Mg^2+^** | **Ca^2+^** | **Cl^-^** | **NO_3_^-^** | **SO_4_^2-^** |
| --- | --- | --- | --- | --- | --- | --- | --- | --- |
| **Na^+^** | 1.00 |  |  |  |  |  |  |  |
| **NH_4_^+^** | -0.13 | 1.00 |  |  |  |  |  |  |
| **K^+^** | 0.36 | 0.44 | 1.00 |  |  |  |  |  |
| **Mg^2+^** | **0.86** | -0.23 | 0.13 | 1.00 |  |  |  |  |
| **Ca^2+^** | **0.72** | -0.31 | -0.02 | **0.81** | 1.00 |  |  |  |
| **Cl^-^** | 0.56 | -0.50 | -0.08 | **0.77** | **0.71** | 1.00 |  |  |
| **NO_3_^-^** | **0.72** | -0.45 | -0.02 | **0.85** | **0.77** | **0.85** | 1.00 |  |
| **SO_4_^2-^** | 0.21 | **0.91** | 0.53 | 0.08 | -0.01 | -0.38 | -0.23 | 1.00 |

| **b)** | **Na^+^** | **NH_4_^+^** | **K^+^** | **Mg^2+^** | **Ca^2+^** | **Cl^-^** | **NO_3_^-^** | **SO_4_^2-^** |
| --- | --- | --- | --- | --- | --- | --- | --- | --- |
| **Na^+^** | 1.00 |  |  |  |  |  |  |  |
| **NH_4_^+^** | -0.02 | 1.00 |  |  |  |  |  |  |
| **K^+^** | 0.67 | 0.44 | 1.00 |  |  |  |  |  |
| **Mg^2+^** | 0.61 | 0.60 | **0.80** | 1.00 |  |  |  |  |
| **Ca^2+^** | 0.69 | -0.28 | 0.40 | 0.50 | 1.00 |  |  |  |
| **Cl^-^** | **0.90** | -0.16 | 0.56 | 0.43 | 0.64 | 1.00 |  |  |
| **NO_3_^-^** | **0.81** | -0.08 | 0.50 | 0.56 | 0.65 | **0.83** | 1.00 |  |
| **SO_4_^2-^** | -0.01 | **0.83** | 0.38 | 0.65 | 0.02 | -0.30 | -0.14 | 1.00 |

SO4^2-^, K^+^, and Mg^2+^ in the tables are non-sea salt fractions. Marked correlations are significant at p<0.01. The range between 0.50 and 0.80 indicates a moderate correlation, while the range between 0.80 and 1.0 suggests a strong correlation.

**Table S2.** Indoor (a) and outdoor (b) metal correlation.

| **(a)** | **Al** | **Fe** | **Cr** | **Mn** | **Co** | **Ni** | **Cu** | **Zn** | **V** | **As** | **Pb** | **Cd** |
| --- | --- | --- | --- | --- | --- | --- | --- | --- | --- | --- | --- | --- |
| **Al** | 1.00 |  |  |  |  |  |  |  |  |  |  |  |
| **Fe** | **0.99** | 1.00 |  |  |  |  |  |  |  |  |  |  |
| **Cr** | **0.95** | **0.91** | 1.00 |  |  |  |  |  |  |  |  |  |
| **Mn** | **0.98** | **0.99** | **0.89** | 1.00 |  |  |  |  |  |  |  |  |
| **Co** | **0.98** | **0.99** | **0.90** | 1.00 | 1.00 |  |  |  |  |  |  |  |
| **Ni** | **0.90** | **0.86** | **0.97** | **0.83** | **0.83** | 1.00 |  |  |  |  |  |  |
| **Cu** | 0.41 | 0.38 | 0.41 | 0.39 | 0.39 | 0.37 | 1.00 |  |  |  |  |  |
| **Zn** | 0.78 | 0.71 | 0.79 | 0.72 | 0.70 | 0.72 | 0.61 | 1.00 |  |  |  |  |
| **V** | **0.80** | **0.82** | 0.77 | **0.80** | **0.80** | **0.83** | 0.36 | 0.55 | 1.00 |  |  |  |
| **As** | **0.80** | 0.74 | **0.90** | 0.70 | 0.70 | **0.95** | 0.31 | 0.65 | 0.66 | 1.00 |  |  |
| **Pb** | 0.74 | 0.75 | 0.74 | 0.74 | 0.74 | 0.78 | 0.43 | 0.50 | 0.78 | 0.72 | 1.00 |  |
| **Cd** | 0.62 | 0.61 | 0.63 | 0.61 | 0.61 | 0.61 | 0.21 | 0.50 | 0.55 | 0.51 | 0.55 | 1.00 |

| **(b)** | **Al** | **Fe** | **Cr** | **Mn** | **Co** | **Ni** | **Cu** | **Zn** | **V** | **As** | **Pb** | **Cd** |
| --- | --- | --- | --- | --- | --- | --- | --- | --- | --- | --- | --- | --- |
| **Al** | 1.00 |  |  |  |  |  |  |  |  |  |  |  |
| **Fe** | **0.99** | 1.00 |  |  |  |  |  |  |  |  |  |  |
| **Cr** | **0.83** | **0.85** | 1.00 |  |  |  |  |  |  |  |  |  |
| **Mn** | **0.98** | **0.99** | **0.84** | 1.00 |  |  |  |  |  |  |  |  |
| **Co** | **0.98** | **0.99** | **0.84** | **0.99** | 1.00 |  |  |  |  |  |  |  |
| **Ni** | 0.62 | 0.64 | **0.91** | 0.62 | 0.63 | 1.00 |  |  |  |  |  |  |
| **Cu** | 0.23 | 0.28 | 0.25 | 0.33 | 0.31 | 0.06 | 1.00 |  |  |  |  |  |
| **Zn** | 0.16 | 0.22 | 0.21 | 0.27 | 0.23 | 0.06 | **0.88** | 1.00 |  |  |  |  |
| **V** | 0.31 | 0.35 | 0.58 | 0.35 | 0.36 | **0.80** | 0.08 | 0.12 | 1.00 |  |  |  |
| **As** | 0.45 | 0.43 | **0.80** | 0.41 | 0.41 | **0.88** | -0.12 | -0.08 | 0.53 | 1.00 |  |  |
| **Pb** | 0.31 | 0.37 | 0.62 | 0.39 | 0.37 | 0.55 | 0.69 | 0.73 | 0.42 | 0.49 | 1.00 |  |
| **Cd** | 0.03 | 0.10 | 0.49 | 0.10 | 0.12 | 0.56 | 0.27 | 0.29 | 0.51 | 0.60 | 0.75 | 1.00 |

Marked correlations are significant at p<0.01. The range between 0.50 and 0.80 indicates a moderate correlation, while the range between 0.80 and 1.0 suggests a strong correlation.
